# Network Diffusion-Constrained Variational Generative Models for Investigating the Molecular Dynamics of Brain Connectomes Under Neurodegeneration

**DOI:** 10.3390/ijms26031062

**Published:** 2025-01-26

**Authors:** Jiajia Xie, Raghav Tandon, Cassie S. Mitchell

**Affiliations:** 1 Laboratory for Pathology Dynamics, Department of Biomedical Engineering, Georgia Institute of Technology and Emory University, Atlanta, GA 30332, USA; 2 Computational Science and Engineering, Georgia Institute of Technology, Atlanta, GA 30332, USA; 3 Machine Learning Center at Georgia Tech, Georgia Institute of Technology, Atlanta, GA 30332, USA

**Keywords:** Alzheimer’s disease, neurodegeneration, machine learning, artificial intelligence, aging, connectome, network decoupling, eigenvalue analysis, pathology dynamics, temporal diffusion network

## Abstract

Alzheimer’s disease (AD) is a complex and progressive neurodegenerative condition with significant societal impact. Understanding the temporal dynamics of its pathology is essential for advancing therapeutic interventions. Empirical and anatomical evidence indicates that network decoupling occurs as a result of gray matter atrophy. However, the scarcity of longitudinal clinical data presents challenges for computer-based simulations. To address this, a first-principles-based, physics-constrained Bayesian framework is proposed to model time-dependent connectome dynamics during neurodegeneration. This temporal diffusion network framework segments pathological progression into discrete time windows and optimizes connectome distributions for biomarker Bayesian regression, conceptualized as a learning problem. The framework employs a variational autoencoder-like architecture with computational enhancements to stabilize and improve training efficiency. Experimental evaluations demonstrate that the proposed temporal meta-models outperform traditional static diffusion models. The models were evaluated using both synthetic and real-world MRI and PET clinical datasets that measure amyloid beta, tau, and glucose metabolism. The framework successfully distinguishes normative aging from AD pathology. Findings provide novel support for the “decoupling” hypothesis and reveal eigenvalue-based evidence of pathological destabilization in AD. Future optimization of the model, integrated with real-world clinical data, is expected to improve applications in personalized medicine for AD and other neurodegenerative diseases.

## 1. Introduction

Alzheimer’s Disease (AD) is currently an incurable disease with a high worldwide societal impact and financial burden [1]. AD is a progressive neurodegenerative disorder characterized by cognitive decline and driven by three key molecular processes: amyloid beta (Aβ) aggregation, **tau** protein dysfunction, and altered glucose metabolism [2,3,4]. The disease begins with the abnormal cleavage of amyloid precursor protein (APP), producing toxic Aβ42 peptides that form soluble oligomers [5,6]. These oligomers disrupt synaptic function and trigger oxidative stress, eventually aggregating into insoluble fibrils and amyloid plaques in the extracellular space. The plaques provoke chronic inflammation, further damaging neurons [7,8,9]. Concurrently, **tau**, a microtubule-associated protein crucial for intracellular transport, becomes hyperphosphorylated, losing its ability to stabilize microtubules [10,11,12]. This leads to **tau** aggregation into neurofibrillary tangles (NFTs) within neurons, impairing axonal transport and spreading across brain regions [13]. The combined Aβ and **tau** molecular pathologies disrupt neural networks and drive neurodegeneration. Glucose metabolism also declines early in AD, with reduced glucose uptake and mitochondrial dysfunction contributing to energy deficits and oxidative stress. This hypometabolism is most prominent in the posterior cingulate cortex and hippocampus and correlates with disease progression [4,14]. Energy deficits amplify Aβ and **tau** pathologies, creating a vicious cycle that exacerbates synaptic loss and neuronal death [15].

### 1.1. The Hypothesized Role of Brain Connectomes in Neurodegeneration

A brain connectome refers to a comprehensive map of neural connections within the brain. It illustrates how different neurons are interconnected through synapses, providing a complete picture of the brain’s structural and functional connectivity at a microscopic level. These molecular dynamics evolve over time: in preclinical stages, Aβ accumulation and subtle metabolic changes occur; in mild cognitive impairment (MCI), plaques and **tau** tangles emerge in key regions, with further metabolic decline; and in Alzheimer’s dementia, widespread pathology and severe energy deficits lead to extensive neurodegeneration [16]. Understanding these interconnected processes provides insight into potential therapeutic targets, such as reducing Aβ aggregation, preventing **tau** phosphorylation, and improving neuronal energy metabolism [17].

Several hypotheses explain the spread of neurodegeneration in AD and are closely related to connectome dynamics:

- Prion-like spread hypothesis—One central hypothesis for the overall molecular dynamics and kinetics of AD is a “prion-like” paradigm caused by the transentorhinal spread and conformational autocatalytic conversion of the misfolded proteins [18]. Aβ and **tau** pathology spreads across the brain via synaptic and axonal connections. This aligns with the concept of network-based propagation, where degeneration cascades through connected nodes in the connectome.
- Network vulnerability hypothesis—Specific brain networks, such as the default mode network (DMN), are more metabolically active and vulnerable to neurodegeneration [19]. Plaques and tangles preferentially accumulate in highly interconnected hubs (e.g., the hippocampus and precuneus), leading to widespread connectivity disruption.
- Synaptic homeostasis hypothesis—Overactive synaptic regions with higher neural activity experience greater amyloid beta release, initiating local neurodegeneration. This disruption spreads through functionally connected areas, degrading network integrity [20].
- Neurovascular hypothesis—Impaired vascular function affects nutrient and waste exchange, leading to hypoxia, inflammation, and plaque accumulation. Connectome regions dependent on efficient blood supply are especially vulnerable, compounding connectivity losses [21].
- Hub vulnerability—Amyloid plaques tend to aggregate in highly interconnected hubs, such as the hippocampus, precuneus, and posterior cingulate cortex [22]. These hubs are crucial for global network integration, and their disruption leads to widespread connectome instability.
- Neuroinflammation and connectome disruption—Aβ accumulation triggers chronic microglial activation, leading to local inflammation [23]. This exacerbates damage to structural and functional connections, creating a feedback loop that amplifies network degradation. Recent research illustrates that treating the inflammatory network with a repurposed anti-inflammatory drug, ketorolac, is promising [24].
- Metabolic stress and network failure—Regions with higher activity and metabolic demands, such as network hubs, are particularly vulnerable to Aβ-induced oxidative stress [23,25]. This results in an impaired energy supply, further disrupting connectivity. This hypothesis also correlates with risk factors from patients with system glucose dysregulation [26]

### 1.2. MRI and PET for Measuring AD Pathology Dynamics

The use of magnetic resonance imaging (MRI) [27] and positron emission tomography (PET) [28] has revolutionized the ability to evaluate disease onset and progression. The development of radiotracers enables the evaluation of hypoperfusion, hypometabolism, neuroinflammation, and amyloid beta deposition [29]. The MRI and PET modalities enable an aggregate measurement of the molecular dynamics, including Aβ aggregation, tau deposition, altered glucose metabolism, and neurodegenerative atrophy [29]. The ability to predict the molecular dynamics spread using standard MRI and PET imaging within a computational framework could improve drug development and personalized medicine. However, a key challenge in the assessment of AD pathology dynamics is the lack of large-scale longitudinal data. The decades-long pathological development of AD further expands the sparsity of longitudinal clinical data [30].

### 1.3. Assessment of AD Pathology Dynamics

Assessing AD pathology dynamics using temporal network models could enable more personalized predictions. Observations from positron emission tomography (PET) scans can be used to track regional distributions of Aβ and tau [31,32]. Network diffusion processes have successfully modeled neurodegenerative-based protein misfolding with results that typically align well with known histopathology [33].

The network-based diffusion mechanism is a system of first-order ODEs. The ODEs are defined by the graph Laplacian as representatives of brain connectomes [33] with additional accumulation, clearance, and propagation (ACP) parameters [31,32,34,35,36,37]. A connectome can be treated as a graph G=(V,E) where the nodes are brain regions, and the edges represent connectivity. Longitudinal validations examining patients across multiple time points have supported the efficacy of the network-based diffusion framework.

Other relevant or related methodologies that have explored the network dynamics of neurodegenerative pathology in the absence of large-scale longitudinal data include the following:

- Static diffusion network models—A major limitation of prior network models is that they typically assumed static brain connectomes and retrieved estimations from healthy and young individuals. Such models ignored the overlapping impacts between normal aging and disease progression that compromise the connectivity of brains [38,39]
- Event base models (EBM)—An EBM uses cross-sectional data to compute various metrics on networks at different stages. A maximum-likelihood estimate determines the ordered sequence in which biomarkers become abnormal [38,40,41]. While an EBM can capture the dynamics of networks, the downstream usage of the sequential aggregated metrics for longitudinal network diffusion modeling is limited by granularity.
- Gaussian process (GP) models—GP models have been constrained by diffusion-related functional dynamics with protein biomarkers at a regional level used as regression variables to infer the connectivity as parameters [30,35,42]. For example, within ACP dynamics, each region can trigger propagation toward connected areas at a maximum rate parameterized by ${\bar{k}}_{i,j}$ sampled jointly with other parameters from a Gaussian process [30,35]. However, these time-independent parameters and the logistically decaying rate are predetermined and handcrafted. Thus, the results are indistinguishable between natural aging and pathological degeneration.
- Physics-informed neural network—Inferring graph dynamics regularized by a physics model is another feasible solution [43] that leverages the advancement of neural ODEs. Although this approach has reached the state of the art, implementation remains challenging without the available network ground truth under neurodegenerative conditions.

### 1.4. Development of Constrained Variational Generative Model to Evaluate Connectome Neurodegeneration Dynamics

**Figure 1:**
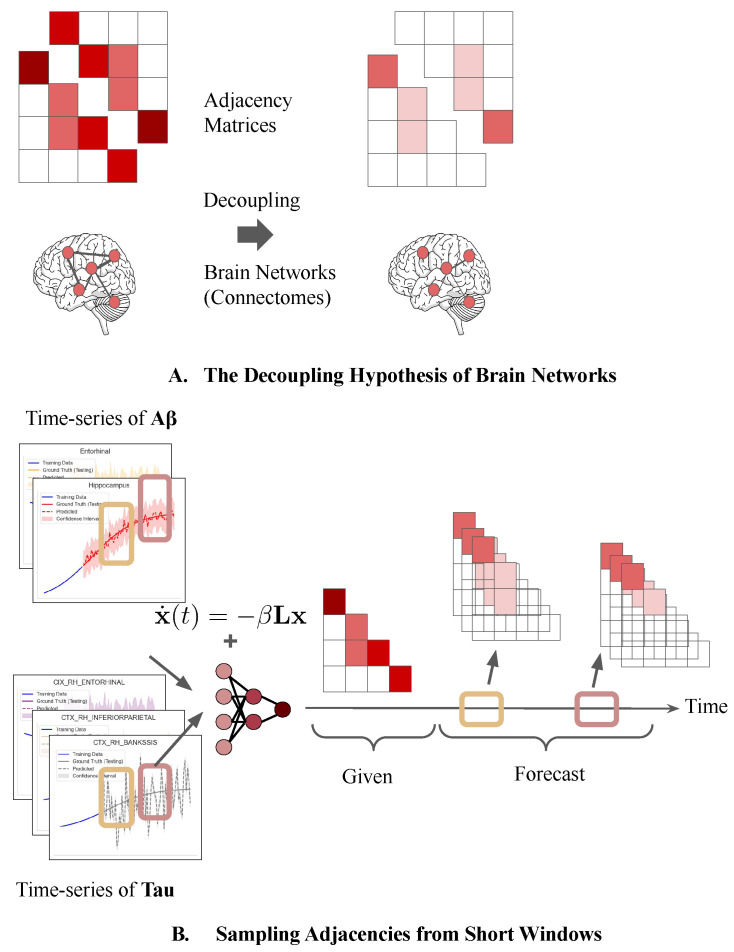
(**A**): The connections among brain connectomes are expected to decouple along the pathology of AD, reflected by the sparsity of adjacency matrices (symmetric and positively semi-definite). No existing longitudinal data can empirically verify this hypothesis. (**B**): It is possible to sample the temporal distributions of the connectomes within short windows of learned constrained generative models given observed time-series biomarkers, assuming the network governing the diffusion process stays constant temporarily within each window. The schematic illustrates a case of 2-windows (“early” and “late”) for the proposed variational generative model.

**Figure 2:**
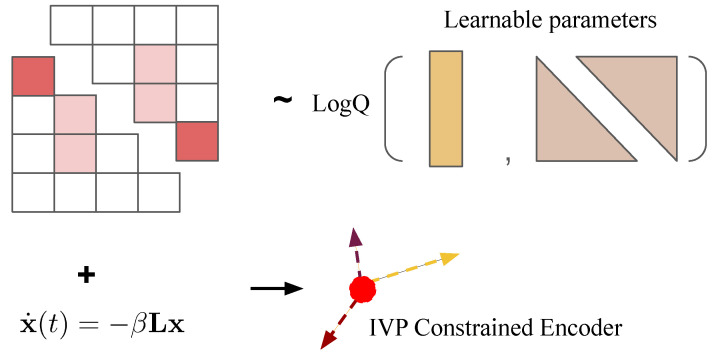
The Bayesian variational inference framework schematic on a short window. The generative process of connectomes for network diffusion models involves an encoder sampling log values normally from a parameterized distribution with a positive definite covariance matrix. It transforms its outer products into adjacency matrices and then into Laplacian matrices. The corresponding decoder is a soft constraint on the optimal conditions of least squares for the uniqueness of the IVP. The effectiveness of this constraint is presented in Figure 3.

The goal of this study was to produce a longitudinally feasible, generalizable, and time-dependent generative process to examine the underlying dynamics of the neurodegenerative connectome. To this end, we derive a first-principles-based, generalizable Bayesian variational inference framework constrained by network diffusion dynamics to generate temporal connectome distributions. Purely with the network diffusion assumption, the framework seeks optimal connectome distributions as a learning problem. It discretizes the long-term pathological dynamics as network dynamics by learning sub-models with connectomes as latent variables and the observed biomarkers as regression variables within a short window (see Figure 1A). Unlike existing studies, this framework makes no prior assumptions on the connectome. Instead, it relies on the parameterized covariance matrix as a Cheolesky decomposition of the variational posterior of a latent vector. Inspired by a variational autoencoder, the generative process involves (1) a decoder from sampled latent variables to reconstruct the input time-series-based biomarkers of Aβ and **tau** with the solutions of the network diffusion models and (2) an encoder to map inputs to the latent space that is additionally constrained by a least-squares condition of the initial value problem (IVP) we derived (see Figure 2). Key contributions of the present work are as follows:

- We propose a generalizable Bayesian variational generative framework capable of estimating temporal connectome (network) dynamics along the progress of neurological disease given a longitudinal biomarker sequence at each window. The latent connectome is modeled by a Gaussian graphical model parameterized by a Cholesky decomposition of the covariance matrix.
- We introduce a novel variational autoencoder framework with the biomarkers as regression variables. The decoder utilizes a single Rayleigh–Ritz pair for an eigenpair approximation to avoid backpropagation on a matrix exponential or a direct eigendecomposition appearing in the solution equations of the network diffusion model. The encoder softly forces the variational distribution of the latent space to a least-squares optimum to guarantee the uniqueness of an IVP.
- Experiments on synthetic and real-world longitudinal datasets of Aβ and **tau** demonstrate (1) the necessity of the soft-constrained encoder; (2) improved long-term regression models with network dynamics retrieved by our framework; and (3) significant evidence of neurodegeneration among AD patients compared to the control group. Notably, network decoupling and instability correlate with disease progression. Collectively, the results indicate that the model quantitatively differentiates pathology from normal aging.

## 2. Results and Discussion

### 2.1. Goals and Setup

The main hypothesis to investigate is the decoupling of edges within brain networks as atrophies, which also indicate disconnections among regions. Given a temporal brain network, the changes in graph adjacency matrices can quantify decoupling. As the networks decouple, the connectivity drops, causing the matrices’ sparsity and decreasing the eigenvalues (see Figure 1A). However, attaining direct longitudinal estimates from human beings is challenging. Nevertheless, constrained variational Bayes models can generate and increment late time-span data (the adjacency matrices). By sampling adjacencies at different timestamps, we can measure the difference among the temporal networks, thereby verifying the decoupling hypothesis in AD pathology (see Figure 1B). Because no ground-truth data are provided, the quality of generated brain networks can only be evaluated based on known clinical knowledge. Given the first-principles methodology with the least assumptions, the generated data should reflect longitudinal and cross-sectional diffusion instability across ages and phases. In other words, misfolded proteins should be less diffusible (unstable) in the late stages of disease and lifespan compared to the early stages. Healthy individuals should be stable and the unhealthy individuals will likely trigger the neurodegeneration pathology of AD. In addition to instability, the generated networks can (1) serve as real-world data to enhance simulation (a.k.a meta-models) for the ACP mechanism of misfolded proteins and (2) be applied to empirically investigate the hypotheses of decoupling in anatomical studies in the future.

The proposed variational Bayesian framework was evaluated by the eigenvalues of the Laplacian matrices, defined as L=D−A, the diagonal degree matrix minus the adjacency matrix which is attained by sampling from the variational posterior. The eigenvalues represent the strength of the instability of the network diffusion kinetics. Additionally, the change in the adjacency matrices provides top-K decoupled pairs of regions. The precision is defined as the true positive pairs divided by K. Details can be found in Section 3.2.

### 2.2. Experiments

Experiments with the Bayesian variational framework were performed to investigate the hypothesis that connectome decoupling appears at different stages of AD on both synthetic and real-world datasets, and the results are presented in this section. By recovering the pathology of connectome degeneration, both novel evidence of neurodegeneration utilizing a generative machine learning algorithm and a feasible, accurate implementation of a simulation for dynamical Laplacian network diffusion can be presented as described in Equation (3). To this end, experimental results comprise three interconnected sections:

- The critical role of the soft constraint of the encoder is first verified by presenting compromised results of the training algorithm when the soft constraint is removed. Because the method lies at the heart of inferring the brain network measured by the reconstruction of the biomarker signals under network diffusion models, the convergence of the training algorithm should be guaranteed.
- The pathological dynamics of connectivity degeneration were investigated through the eigenvalues of the graph Laplacian, which also provides insights into the instability of the brain’s dynamical systems both longitudinally and cross-sectionally. The cross-sectional study matches three stages, healthy (H), mild cognitive impairment (MCI), and AD, among participants to differentiate the stages of disease. The longitudinal study explores the decay rate of the same participants from early to late stages.
- Enhanced results of Equation (3) are presented, suggesting that the inferred network dynamics can improve long-term longitudinal simulations for future studies.
- The designed approach is then utilized to identify which pairs of brains contribute to neurodegeneration under AD perturbation. Because the magnitude of decoupling is unknown, a synthetic dataset of biomarkers is created by reducing the values of hypothesized pairs on the graph adjacency and feeding it into the simulation model of network diffusion. The difference in the output between the generated graph adjacencies should reflect the exact change in pairs. With the above sanity check, the model is applied with real-world data to identify which pairs decayed the most in AD.

### 2.3. Soft Constraints as the Novel Encoder Improve Regression Error and Justify Study Approach

**Figure 3:**
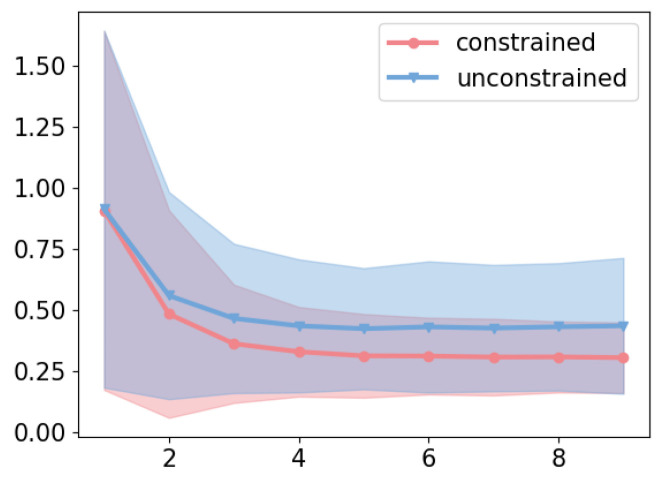
The loss of fit without/with the soft constraint defined in Theorem 2. The X-axis is epochs (each with 100 samples) × 100.

The network diffusion kinetics assumption is vital for the model-to-data methodology. The generative model we designed learns the best network representation from the network diffusion equation by fitting the solution equation to the time series of biomarkers. The regression error must serve as the key justification for the method from a domain standpoint. Therefore, we need to reduce the regression error, e.g., mean-squared error (MSE), to be as low as possible. However, the underdetermined IVP has been a significant bottleneck in controlling the MSE, given that our method utilizes matrices’ lower-rank approximation to simplify the training numerical algorithm and solve the network diffusion ODE. To this end, we propose an encoder to softly constrain the latent variable as close to the least-squares optimal solution as possible. To validate this novel approach, we first present the results of an ablation study on all datasets by removing the soft constraint for the uniqueness of IVPs. Figure 3 suggests slower and inferior convergence of the reconstruction loss, also known as the regression error, by removing the soft constraint on the variational posterior. Without the constraint, the model was under-fitted to the biomarkers. Intuitively, the soft constraint turns the IVP problem into a least-squares problem that has a unique optimal solution. The constraint benefits the model by regularizing the parameters into the space that is originally close enough to the original least-squares problem. This recapitulates the necessity of soft constraints for inferring the latent connectome measured critically by the goodness of fit.

### 2.4. The Proposed Framework Can Differentiate Network Dynamics Longitudinally and Cross-Sectionally

**Figure 4:**
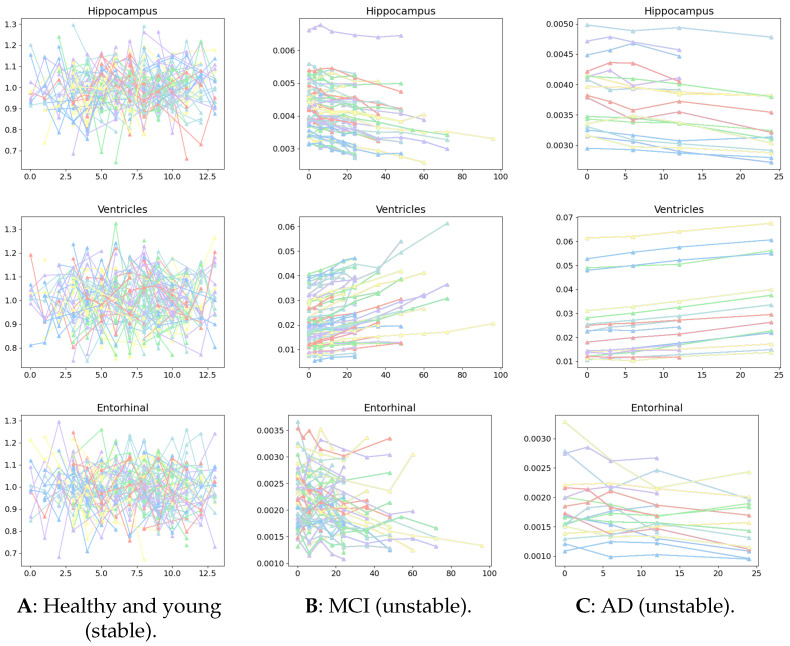
The Aβ time-series pathological biomarkers of three regions: hippocampus, ventricles, and entorhinals. Each color indicates an individual with all longitudinal observations. (**A**): Healthy and young Synthetic-AV45 individuals are considered stable in terms of small eigenvalues of graph Laplacian; (**B**): MCI and (**C**): AD ADNI-AV45-PET individuals are unstable, corresponding to larger eigenvalues. The eigenvalues inferred by our model can be found in Figure 5.

A significant extension of our framework is that it can differentiate network dynamics between (healthy and young), (healthy and old), MCI, and AD stages from the Aβ biomarker time series. The healthy and young cohort without normal aging is represented by Synthetic-AV45, where the healthy and young stage appears as random white noise in Figure 4A, compared against Figure 4B, as a mixed impact between normal aging and pathology. The main difference between them is that Figure 4A is considered stable and Figure 4B is unstable. The stability is measured by the eigenvalues of the graph Laplacian which is the momentum of Aβ diffusing across the brain network. We observed that each sub-figure of Figure 4 has a different momentum to trigger a diffusion mechanism. We will show that the inferred largest eigenvalues of the graph Laplacian directly inform this message, and the changes are consistent with the stages of AD and age.

**Figure 5:**
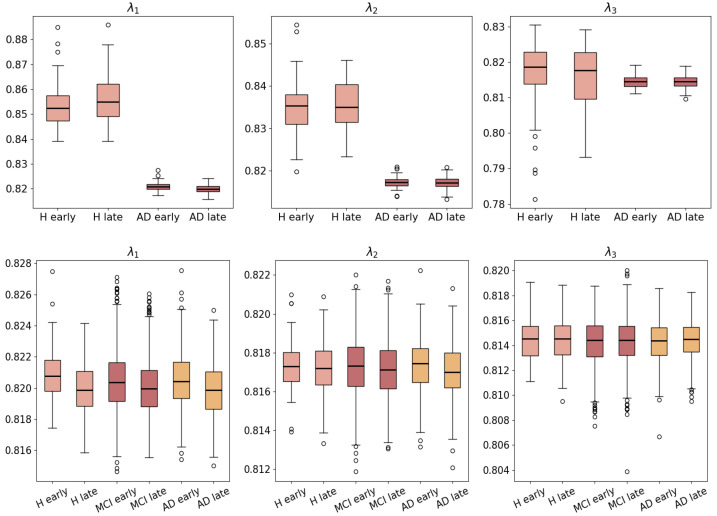
(**Top**): The top 3 eigenvalues of the inferred graph Laplacian between healthy (young) and AD Synthetic-AV45 individuals. (**Bottom**): The top 3 eigenvalues of the inferred graph Laplacian from healthy (old) and MCI to AD ADNI-AV45-PET individuals.

According to Figure 5(Top) of ADNI-AV45-PET and Figure 5(Bottom) of Synthetic-AV45, our method presents distinguishable degeneration in the top three eigenvalues among network dynamics from the “early” stage to the “late” stage of MCI and AD compared to H. First, according to Figure 5(Bottom), for every “early” and “late” pair, H (healthy and young), MCI, and AD’s eigenvalues in the “early” stage are always larger than those in the corresponding “late” stage. In contrast, reflected by the network diffusion models, the eigenvalues of the healthy and young cohort have no significant difference from the “early” to the “late” stage, according to Figure 5(Top), which is synonymous with normal physiological homeostasis. This suggests that the effect of normal aging is more obvious within the stages of AD, MCI, and H (healthy and old). Comparing the “late” stage’s eigenvalues, the difference becomes unclear because the end stage is overlapped between normal aging and disease. Nevertheless, it is still feasible to track the pathology dynamics across different stages. Since the cohort of H (healthy and old) in Figure 5(Bottom) represents the force of normal aging, the “early” stage’s eigenvalues of MCI and AD are lower than their counterparts in H and MCI, showing quantitative evidence of acceleration caused by the disease.

### 2.5. Meta Network Diffusion Models with Inferred Dynamical Networks from Multiple Short Windows Are More Realistic

**Figure 6:**
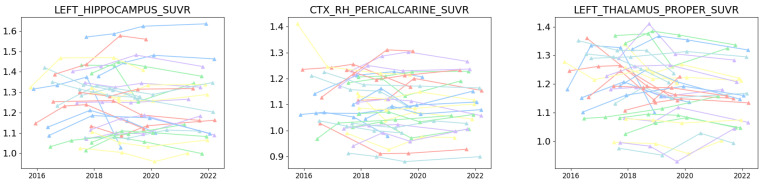
The **tau** time-series pathological biomarkers of three sampled regions out of 82 regions from ADNI-1451-PET. Unlike ADNI-AV45-PET, the Aβ dataset, ADNI-1451-PET has no labels to categorize H, MCI, and AD.

**Figure 7:**
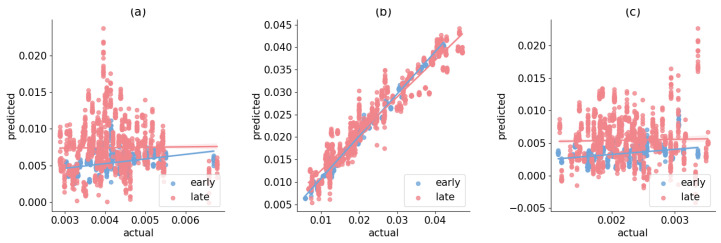
The optimal meta-model’s (no-source 2-networks) regression results in the hippocampus (**a**), ventricles (**b**), and entorhinal (**c**) from MCI and AD individuals of Adni-AV45-PET.

We compare meta-models with different ACPs to show that a dynamical network diffusion model can outperform a typical network diffusion model with static networks. In addition to the Aβ datasets, the **tau** time-series dataset ADNI-1451-PET is added to analyze a higher-dimension scenario (82 regions vs. 4). See Figure 6 for an illustration of the three sampled regions. Since our approach can approximate the network distribution given any short-window times series, we can at least attain two distributions, one for the “early” and the other for the “late” stage, respectively. We show that the regression results will improve with the network dynamics of the two networks compared to the meta-model with a static network. Table 1 presents the MSE of the meta-models by calibrating its meta parameters β, ξ, r, and a. The MSEs of the 2-windows dynamical systems are lower than the static network diffusion model. This suggests that we can potentially infer the network dynamics with more short windows and implement the discretized Equation (3) with further refinement of the ACP terms [35]. The improvement in the meta-models is due to the explained variance by differentiating the degree of longitudinal decoupling (Figure 7). As seen in Table 2, the Pearson correlation coefficients between the actual and the predicted by the meta-models are all significant in the early stage given the inferred graph Laplacian in the first time window. The biomarkers (regression variables) versus the actual “early” stage of MCI and AD have a lower deviation from the line in the three regions, indicating better-explained variance. The “early” stage has large eigenvalues (Figure 5(Top, Bottom)), corresponding to the strength of the diffusion process. In contrast, the “late” stage illustrates network decoupling with amplified variance, as suggested empirically by [44]. With the two inferred networks, a single meta-model can factor out the impact between time and neurodegeneration, which benefits future predictive quantitative simulations.

### 2.6. The Variational Bayesian Framework Outperforms the Discrete EBM Model in Detecting Atrophy as Decoupling Among Subcortical Regions

**Figure 8:**
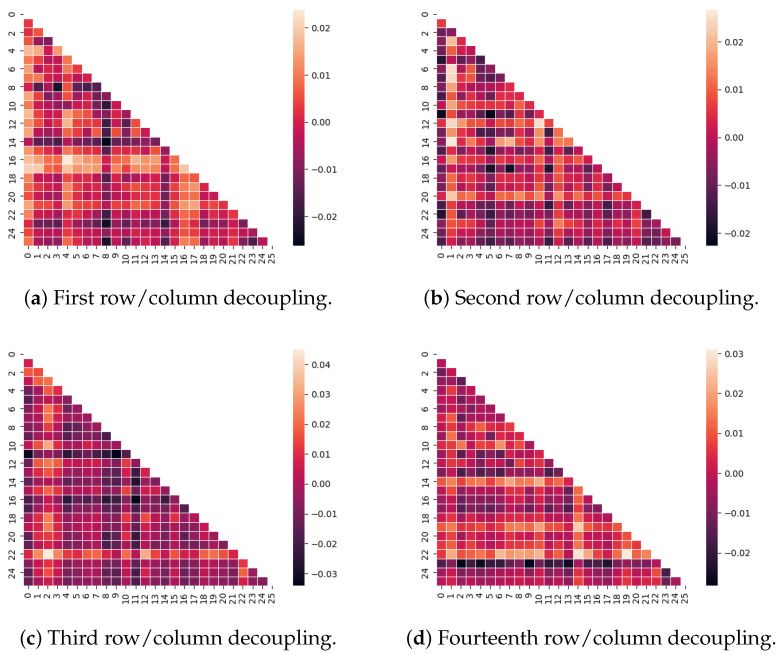
The difference matrices attained from the 2-windows model (before–later) using the synthetic data Synthetic-1451-PET experiments, where we picked a column and row from the graph adjacency matrices and reduced the value of the cells by 0.02. For better visualization, we picked the rows and columns from index 30 to index 45 (26 areas total). Each row/column with consistently brighter cell colors is considered an atrophied area.

**Figure 9:**
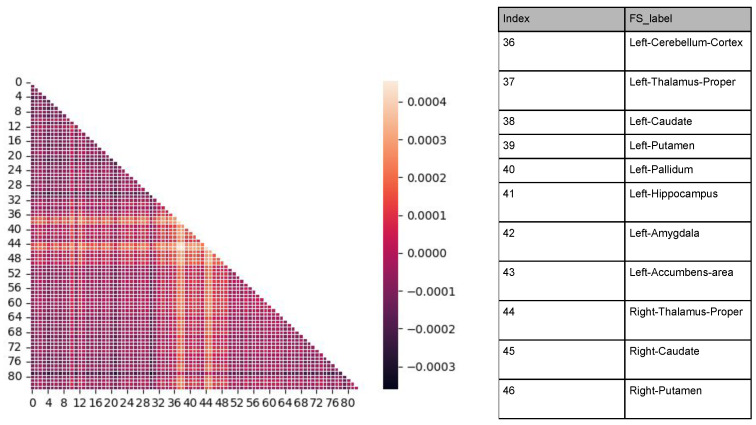
The difference (early–late) of the adjacency matrices between the two stages from Adni-1451-PET.

According to Table 3, the averaged precision and its standard deviation attained from the largest 10 cells on the difference matrix from the 2-windows model is better than taking the leading 10 values from the sequence yielded by an EBM model. The decoupling pairs among the inferred adjacency matrices from the synthetic dataset *Synthetic-1451-PET* are visualized where the 1st, 2nd, and 14th rows and columns decay by 0.02. Based on Figure 8a–d, each row/column with consistently brighter colors of cells is considered an atrophied area. The generated graph adjacency approximately reflects the changes in the cells with the correct magnitude, followed by a subset of false positives. This suggests that the models have factored out every positive ground truth, i.e., a 100% recall. The remaining problem is to control the amount of false positives by optimizing a threshold. Based on the success of the experiment, *Adni-1451-Pet* was applied as a case study to visualize the decoupling from the adjacency matrix. Figure 9 highlights the areas of the brain suffering from decoupling mostly between index 36 (cerebellum cortex) and index 45 (accumbens). These highlighted subcortical areas shown in the table within Figure 9 suffer from various degrees of atrophy in the pathology of MCI and AD [45].

### 2.7. Comparative Context with Prior Work Examining Neurodegenerative Dynamics

Prior AD work has shown that the temporal progression of pathological changes follows a characteristic pattern across brain regions, aligning with the prion-like spread of **tau** pathology and connectome vulnerabilities [18]. Initially, **tau** aggregates emerge in the entorhinal cortex and hippocampus, regions critical for memory, and progressively spread to the amygdala and other limbic structures [16]. As the disease advances, cortical areas, including the cerebral cortex, exhibit significant atrophy, particularly in association and posterior regions. Subcortical structures such as the thalamus, caudate, putamen, pallidum, and nucleus accumbens are later affected [46], with varying degrees of vulnerability influenced by their connectivity profiles. The connectome’s organization, with hub regions being highly interconnected, makes these areas particularly susceptible to degeneration [47]. Thus, the computational model predicted results of Figure 9 are supported by the literature.

Interestingly, unstable dynamics have been identified in other models of neurodegeneration, including secondary spinal cord injury [48] and amyotrophic lateral sclerosis (ALS) [49,50]. A recent ODE feedback model used transgenic SOD1-G93A ALS mouse data and eigenvalues to identify a mathematical instability in ALS regulatory dynamics [50]. In silico combination treatments specifically focused on stabilizing the system were found to be most successful in countering disease progression [50].

Likewise, the present work used eigenvalues to illustrate the instability of the (Aβ) processes. Instability increased with age and MCI in particular. While some level of (Aβ) instability is likely intertwined with aging, clearly, greater instability is seen with MCI and especially AD. This trend further suggests that system instability is critical to disease progression. Amplified variance in late-identified stages of cerebrospinal fluid peptides [44] and oscillations in proteins [51] suggest a regulatory attempt to bring a highly unstable pathophysiological system closer to homeostasis. Much like the work of Lee and colleagues studying ALS [50], the present ODE-based AD clinical progression model could potentially be adapted to evaluate therapies that bring the underlying dynamics back to a more stable, healthy state.

Other existing empirical and anatomical studies show that neurodegenerative diseases have complex mechanisms with multi-scalar dynamics. As the grey matter atrophies, there is an anatomical network decoupling of brain regions with different stages of symptomatic dementia [38,52,53,54,55,56,57]. The present study corroborates the presence of network decoupling via the visualized adjacency matrices. Future work may also be able to use modeled differences in network dynamics to differentiate overlapping disease processes, such as dementia associated with ALS, Parkinson’s disease, supranuclear palsy, and corticobasal syndrome [58].

### 2.8. Limitations

The inferred networks have three limitations that warrant future work. (1) Consistency: It was observed that the difference between the “early” and the “late” windows can be negative, indicating a strengthened connection. A decoupled connection cannot be re-established in the future, which challenges the current framework to consider a constraint or other alternative approach to the short windows. (2) Overfitting: Experimental results may become insignificant with over-training on each short window. Overfitting will result in numerical errors dominating the adjacency matrices. Future work is necessary to identify optimal stopping criteria and a new optimization formulation or optimal condition to replace the current training loss. (3) A robust estimation of each short window needs over-sampling from the variational posterior at every training iteration. The sampling algorithm becomes inefficient with higher dimensions. Future work will address the scalability of the framework. (4) False positives: Our generative approach tends to have false positive decoupling pairs, as seen in Figure 8. The current framework cannot control and trade off the decision boundary purely according to the difference matrices. Future work should address this limitation by either seeking prior knowledge or sparsity constraints, e.g., Lasso, as regularization to the parameters.

## 3. Materials and Methods

The methods of this study are described, including data sources, experimental metrics, experimental design, the integrative framework, and corresponding mathematical proofs.

### 3.1. Data Sources

The Alzheimer’s Disease Neuroimaging Initiative (ADNI) was launched as a public–private partnership, led by Principal Investigator Michael W. Weiner, MD. The primary goal of ADNI is to test whether serial magnetic resonance imaging (MRI), PET, other biological markers, and assessments can be combined to measure the progression of MCI and early AD. For up-to-date information, see www.adni-info.org, accessed on 28 December 2024. (Data used in the preparation of this article were obtained from the Alzheimer’s Disease Neuroimaging Initiative (ADNI) database (adni.loni.usc.edu), accessed on 28 December 2024. As such, the investigators within the ADNI contributed to the design and implementation of ADNI and/or provided data but did not participate in the analysis or writing of this report.) For the experiments, two real-world regional summarized datasets were applied, plus a synthetic dataset:

1. ADNI-AV45-PET: A dataset collected from PET scans tracing the Aβ protein among 770 participants from ADNI with 1477 longitudinal data points. These data were used by Garbarino et al. [30,35]. The participants are categorized as H, MCI, and AD. We used a condensed version aggregated into 4 parts of the brain: hippocampus, ventricles, entorhinal, and others.
2. ADNI-1451-PET: These regional summary flortaucipir data of over 82 brain areas for tracing **tau** distribution were used by Thompson et al. [39]; they provide mean **tau** PET intensity values for each of the regions in the Desikan–Killiany atlas, over 134 participants.
3. Synthetic-AV45: There is not a large cohort of participants for longitudinal validation. In particular, there is a shortage of healthy, young participants, and longitudinal AD data. Therefore, we created a synthetic experimental dataset for simulating ADNI-AV45-PET. We added random noise to 4D logistic-like curves in the “late” stage to create the dataset for simulating AD biomarkers and add noise to flat curves for simulating healthy and young participants’ biomarkers. The flatter curves are based on the assumption of stable physiological homeostasis in a young, healthy population.
4. Synthetic-1451-PET: There are no ground-truth data with labels of the exact magnitude of regeneration on the connectome networks. The original graph adjacency matrix is perturbed by $A\left(t\right)=A_{0}-0.02t$ on a column we picked, where t∈[0,1]. Random noise was added to the curve derived from the solution function of the network diffusion ODE $f(t|x_{0},\beta ,L\left(t\right))+\delta$ to attained the synthetic biomarkers.

### 3.2. Metrics

The predicted network dynamics cannot be evaluated in terms of standard error metrics given no ground-truth data are available. Instead, the measure of success is a clear distinction between decoupling among different disease stages (H, MCI, and AD). To measure the degeneration, we propose using the top-K eigenvalues of the inferred graph Laplacian. There is theoretical intuition for this metric here. Namely, if all eigenvalues of the graph Laplacian are non-negative, the fixed points of any dynamic network diffusion from Equation (3) are unstable, assuming the source term is non-negative too. Normally, a participant will experience neurodegeneration as a mixture of the impact between aging and neurological disease. Each realization of $L_{t}$ in a short window is independent of time. It represents the force/momentum to converge to the next stage of the disease $L_{t+1}$ purely with the network diffusion kinetics under small perturbation.

Another metric used to compare against the EBM is the precision, defined as the number of true positives divided by the number of predicted positives. The predicted positives depend on the top-K values selected. We picked K=10. Meanwhile, the method was evaluated by the regression error of the meta-models based on discretized Equation (3) with the inferred networks at each window.

### 3.3. Longitudinal Experiment Design

Because the major researched participants have no more than 5 longitudinal data points, the timestamped data were divided evenly into “early” and “late” groups. For the meta-model, two scenarios are compared: (1) 1-Network: the meta-model uses the same $L_{t_{1}}$ inferred from the “early” stage window; (2) 2-Network: the meta-model uses $L_{t_{1}}$ inferred from the “early” stage window and $L_{t_{2}}$ inferred from the “late” stage window.

### 3.4. Meta Models

After inferring the sequence of $L_{t_{1}}$ and $L_{t_{2}}$, we fit meta-models of Equation (3) with three different source terms representing variations of the ACP mechanism [32]: (1) a zero-source term; (2) a linear-source term, s(t)=rt; and an exponential source term, $s\left(t\right)=a\left(e^{\xi t}-1\right)$. We show that the regression results will be improved with the network dynamics of the two networks $L_{t_{1}},L_{t_{2}}$ compared to the meta-model with a single static $L_{t_{1}}$. Both lead to in-homogeneous ODE systems. The solution equation of Equation (3) with the linear and exponential source is as follows:

(1) $$\begin{array}{cc} \; & x\left(t\right)\approx Ue^{-\beta t\Lambda }U^{⊤}x_{0}+\\ \; & Ue^{-\beta t\Lambda }U^{⊤}r\left(\frac{1}{\beta \lambda_{1}}e^{\beta t\lambda_{1}}-\frac{1}{\beta^{2}\lambda_{1}^{2}}\left(e_{1}^{\beta t\lambda }-1\right)\right) \end{array}$$

(2) $$\begin{array}{cc} \; & x\left(t\right)\approx Ue^{-\beta t\Lambda }U^{⊤}x_{0}+\\ \; & Ue^{-\beta t\Lambda }U^{⊤}a(\frac{1}{\beta \lambda_{1}+\xi }\left(e^{t(\beta \lambda_{1}+\xi )}-1\right)\\ \; & -\frac{1}{\beta \lambda_{1}}\left(e^{\beta t\lambda_{1}}-1\right)) \end{array}$$

### 3.5. Dynamical Network Diffusion Models and ACP

We consider a brain consisting of *N* interconnecting regions, equivalently represented by a network G=(V,E). The main hypothesis investigated is that the brain connectome has dynamics dependent on *t* as well, denoted by G(t). Let A(t) be the corresponding adjacency matrix and L(t)=D(t)−A(t) be the corresponding graph Laplacian, defined as the degree matrix minus the adjacency matrix. We adopted the notations from Yang et al. [32]. A dynamic network diffusion process is a system of first-order ODEs with respect to time *t* defined as

(3) $$\dot{x}\left(t\right)=-t\beta L\left(t\right)x+s\left(t\right)$$

where β and s(t) are parameters. The solution x(t) is an *N*-dimensional vector defining the dynamics of **tau** or Aβ concentrations at each region. An ACP mechanism can be modeled with different source terms of s(t), e.g., linear or exponential [32]. A discrete realization of the above equation requires longitudinal estimates of $L_{1},L_{2}\ldots L_{T}$, which is hard and expensive to retrieve. The goal is to infer these connectomes in sequential order.

### 3.6. Overview of Proposed Model Solution

**Figure 10:**
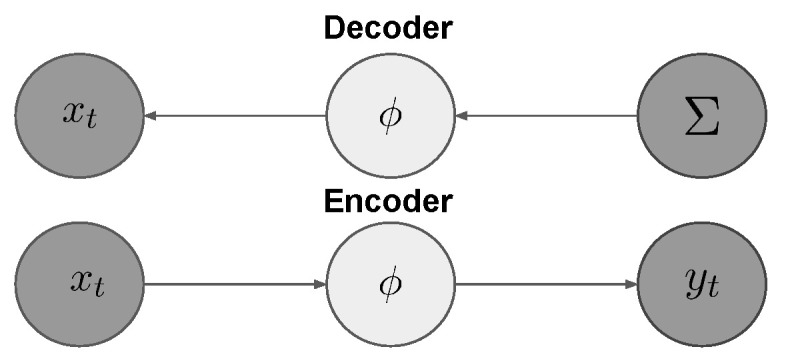
The Bayesian variational autoencoder. $x_{t}$ represents the time series points of the misfolded protein. $y_{t}$ represents the projected initial value observations. Σ is the covariance matrix of the prior. ϕ is the latent variable.

We tackle the problem with a generalizable variational autoencoder framework to infer connectomes within a short window. Both the decoder and encoder are connected by a latent variable for modeling the covariance matrix of the Gaussian graphical model (see Figure 10). In this section, the decoder and encoder are described in detail.

### 3.7. Decoder

We assume that within each short window, a sequence of $\left\{x_{0},x_{1}\ldots x_{T}\right\}$ is obtained. The key methodology we propose is to take the posterior of connectomes within each window as independent of time to be inferred from the variational generative process. The decoder is a Gaussian graphical model with a given prior parameter of the covariance matrix Σ, which can be estimated from healthy and young participants, as in [39]. The matrix Σ must be symmetric, positive, and definite, as per the definition of undirected graph adjacency. We assume a latent variable ϕ is sampled from a multivariate log-normal distribution logϕ∼N(0,Σ). Following the Gaussian graphical model, we model the graph Laplacian defined as

(4) $$\begin{array}{c} A=\phi \phi^{⊤}-\mathrm{Diag}\left(\phi \phi^{⊤}\right),\;L=\sum_{i=1}^{N}{\left(A1\right)}_{i}e_{i}e_{i}^{⊤}-A \end{array}$$

The log-normal distribution ensures non-negative cells of the adjacency matrix *A* required by the definition.

**Theorem 1.**  *L is a graph Laplacian with a zero eigenvalue corresponding to the eigenvector 1 [59].*

With L as the latent variable uniquely defined by ϕ, each observation $x_{t}$ for 1≤t≤T is modeled by a regression function given by

(5) $$x_{t}=f\left(t|x_{0},\beta ,L\right)+\epsilon$$

where ϵ is Gaussian noise and $f(t|x_{0},\beta ,L)$ is the solution of $\dot{x}\left(t\right)=-t\beta Lx$. Note that we remove the source term s(t) as in Equation (3) because the diffusion process dominates clearance and generation in the short term [30]. The above equation has a closed-form solution for an initial value problem given by

(6) $$f\left(t|x_{0},\beta ,L\right)=e^{-t\beta L}x_{0}$$

#### Stabilizing Gradient and Numerical Approximation

The above forward process will likely encounter gradient explosion because the matrix exponential inherits the exponential of the latent vector $\phi =e^{\log\phi }$. We address this problem first with the eigendecomposition of $L=U\Lambda U^{⊤}$, which decomposes the matrix exponential in the solution as well:

(7) $$f\left(t|x_{0},\beta ,L\right)=\sum_{i=1}^{N}e^{-t\beta \lambda_{i}}u_{i}^{⊤}x_{0}u_{i}$$

A nice property of the eigenvalue of L is the upper bound in $O(\frac{N}{N-1})$ [59]. However, computing the eigendecomposition for every sample and iterative backpropagation can be expensive. We can consider the largest eigenvalue and eigenvector as a rank-1 approximation. For the best efficiency, we recommend using unitized ϕ as a 1-D Rayleitz–Ritz vector for approximating an eigen-pair of the graph Laplacian. That is, we approximate an eigenvalue as

(8) $$\bar{\lambda }=\frac{\phi^{⊤}L\phi }{\phi^{⊤}\phi }=\frac{\sum_{i<j}\phi_{i}\phi_{j}{\left(\phi_{i}-\phi_{j}\right)}^{2}}{\phi^{⊤}\phi }$$

The approximated solution equation is therefore simplified:

(9) $$f\left(t|x_{0},\beta ,L\right)=e^{-t\beta \bar{\lambda }}\frac{\phi^{⊤}x_{0}}{\phi^{⊤}\phi }\phi$$

### 3.8. Encoder

The encoder differs from a typical encoder of variational autoencoders, which map from the space of $x_{t}$ to the latent space of ϕ. Because Equation (9) is based on a rank-1 matrix, the solution space for the IVP is large. To see this, note that the orthogonal matrix from Equation (7) has a unique IVP, $U^{⊤}x_{0}$, compared to $\phi^{⊤}x_{0}$. This underdetermined property will cause an ill-conditioned optimization problem during the training process compromising the convergence, which we will show later in an ablation study. We address this issue through a least-squares optimal constraint on the latent space of ϕ. Observe that any ϕ with $x_{0}^{⊤}$ multiplying to the left of Equation (9) yields

(10) $$x_{0}^{⊤}x\left(t\right)=e^{-t\beta \bar{\lambda }}\frac{{\left(\phi^{⊤}x_{0}\right)}^{2}}{\phi^{⊤}\phi }$$

The intuition is based on the set of sampled $\phi^{1},\phi^{2}\ldots \phi^{M}$ attained in the training process through sampling from the variational posterior because, for each learning problem, they share the same $x_{0}$. If *M* is properly chosen, they can be combined to form a basis to ensure the uniqueness of the solution. If *M* is large, it will lead to an over-determined system. To see this, consider a sample $\phi^{k}$ from k=1,2…M for the same $x_{0}$ window. For any $x_{t}$, the least-squares Problem 11 is given by

(11) $$\min\sum_{k=1}^{M}{\left(x_{0}^{⊤}x_{t}-e^{-t\beta \bar{\lambda }}\frac{{\left({\phi^{k}}^{⊤}x_{0}\right)}^{2}}{{\phi^{k}}^{⊤}\phi^{k}}\right)}^{2}$$

**Theorem 2.**  *Problem 11 has an optimal solution when for every k=1…M.*

$$x_{0}^{⊤}x_{t}=\frac{{\left({\phi^{k}}^{⊤}x_{t}\right)}^{2}}{{\phi^{k}}^{⊤}\phi^{k}}$$

A benefit of the above condition is it eliminates the exponential term. Therefore, the optimal condition constraint is applied as another regression variable in the decoder. That is, we let $y_{t}=x_{0}^{⊤}x_{t}$ be the observed value of the constraint and the regression problem is

(12) $$y_{t}=\frac{{\left(\phi^{⊤}x_{t}\right)}^{2}}{\phi^{⊤}\phi }+\epsilon$$

The aforementioned soft constraint corresponds to the decoder specifically designed for this study, as schematically illustrated in Figure 10.

### 3.9. Learning Problem and Training Objective

We now integrate the encoder and decoder to formulate the learning problem. Recall that the encoder first samples latent vector ϕ from the log-normal prior. Deriving a closed-form posterior remains impossible for the over-complicated formulation of the graph Laplacian. We therefore parameterize a variational posterior as

(13) $$q\left(\log\phi \right)=N\left(\nu ,\Psi \Psi^{⊤}\right)$$

where ν is an *N*-dimensional vector and Ψ is a lower-triangular matrix with a positive diagonal. The positive diagonal constraint ensures a symmetric and positive definite covariance matrix and it also serves as the Cholesky factor for the reparameterization trick. For δ∼N(0,I), a new sample is generated by

(14) logϕ=ν+Ψδ

To avoid constrained optimization, we simply set the diagonal components of Ψ as positive constants and leave all off-diagonal parts as parameters to learn.

We start with the likelihood given pair of ($x_{t},x_{0}$) and the observed constraint $y_{t}$ to formulate the learning objective:

(15) $$\begin{array}{c} p\left(x_{t},y_{t}|f,x_{0},\beta \right)=\int p\left(x_{t}|\phi ,f,x_{0},\beta \right)\\ q\left(y_{t}|\phi ,x_{t},x_{0}\right)p\left(\log\phi |\Sigma \right)d\log\phi \end{array}$$

Using the evidence lower bound of the above likelihood, the training objective is

(16) $$\begin{array}{c} L\left(\Psi ,\nu \right)=E_{q(\log\phi )}\log\left(p\left(x_{t}|\phi ,f,x_{0},\beta \right)q\left(y_{t}|\phi ,x_{t},x_{0}\right)\right)\\ -D_{\mathrm{KL}}\left(q(\log\phi )∥p(\log\phi |\Sigma )\right) \end{array}$$

The learning problem is $\max_{\Psi ,\nu }L\left(\Psi ,\nu \right)$. For each window $t_{1}$, $t_{2},\ldots$ and $t_{T}$, we train for the optimal Ψ,ν. Then, the adjacency matrix for each window can be estimated by first sampling based on Equation (14), then averaging the normalized outer products:

(17) $$A_{t}\approx \frac{1}{M}\sum_{k=1}^{M}\frac{\phi^{k}{\phi^{k}}^{⊤}}{{\phi^{k}}^{⊤}\phi^{k}}$$

We leave β as a hyper-parameter to be fine-tuned when $L_{t_{1}}$, $L_{t_{2}},\ldots$, $L_{T}$ is ready for implementing the discretized meta-model of Equation (3).

### 3.10. Proof of Theorem 1

**Proof.** 
$$\begin{array}{cc} \; & L1=\sum_{i=1}^{N}{\left(A1\right)}_{i}e_{i}-\phi \left(\sum_{i=1}^{N}\phi_{i}\right)+\phi ⊙\phi \\ \; & =\phi \left(\sum_{i=1}^{N}\phi_{i}\right)-\phi ⊙\phi -\phi \left(\sum_{i=1}^{N}\phi_{i}\right)+\phi ⊙\phi =0 \end{array}$$

□

### 3.11. Proof of Theorem 2

**Proof.**  First, note that $\dot{x}\left(t\right)=-t\beta Lx$ is Lipschitz-continuous in x:

$$∥-t\beta L\left(x_{1}-x_{2}\right)∥\leq |t\beta |∥L∥∥x_{1}-x_{2}∥$$

As per the Picard–Lindelöf theorem [60], for every ($x_{0},x_{t}$) pair, the IVP problem within a time interval containing the two points is unique, and for our case, it is given by

$$x_{t}=e^{-t\beta L}x_{0}$$

Due to the uniqueness, reverse dynamics with $x_{t}$ as the IVP must exist and is given by

$$x_{0}=e^{t=0\beta L}x_{t}$$

Then, applying the same Rayleitz–Ritz approximation, the following equality must hold:

$$\frac{{\left(\phi^{⊤}x_{t}\right)}^{2}}{\phi^{⊤}\phi }=x_{0}^{⊤}x_{t}=e^{-t\beta \bar{\lambda }}\frac{{\left(\phi^{⊤}x_{0}\right)}^{2}}{\phi^{⊤}\phi }$$

□

### 3.12. Derivation of Rayleitz–Ritz Value in Equation (8)

We break down the graphical Laplacian as the degree matrix minus the adjacency matrix:

$$\begin{array}{cc} \; & \frac{\phi^{⊤}A\phi }{\phi^{⊤}\phi }=\frac{{\left(\phi^{⊤}\phi \right)}^{2}-\phi^{⊤}\mathrm{Diag}\left(\phi \phi^{⊤}\right)\phi }{\phi^{⊤}\phi }\\ \; & ={∥\phi ∥}^{2}-\frac{\sum_{i=1}^{N}\phi_{i}^{4}}{{∥\phi ∥}^{2}} \end{array}$$

$$\begin{array}{cc} \; & \frac{\phi^{⊤}\left(\sum_{i=1}^{N}{\left(A1\right)}_{i}e_{i}e_{i}^{⊤}\right)\phi }{\phi^{⊤}\phi }=\sum_{i=1}^{N}{\left(A1\right)}_{i}\frac{\phi_{i}^{2}}{{∥\phi ∥}^{2}}\\ \; & =\sum_{i=1}^{N}\left(\phi^{⊤}1\phi_{i}-\phi_{i}^{2}\right)\frac{\phi_{i}^{2}}{{∥\phi ∥}^{2}}\\ \; & =\sum_{i=1}^{N}\phi^{⊤}1\frac{\phi_{i}^{3}}{{∥\phi ∥}^{2}}-\frac{\sum_{i=1}^{N}\phi_{i}^{4}}{{∥\phi ∥}^{2}} \end{array}$$

Therefore, we combine these two terms together:

ϕ⊤Lϕϕ⊤ϕ=ϕ⊤(∑i=1NA1ieiei⊤)−Aϕϕ⊤ϕ=∑i=1Nϕ⊤1ϕi3∥ϕ∥2−∑i=1Nϕi4∥ϕ∥2−∥ϕ∥2+∑i=1Nϕi4∥ϕ∥2=∑i=1N∑j=1Nϕiϕj3∥ϕ∥2−∥ϕ∥2=∑i=1N∑j=1Nϕiϕj3−ϕi2ϕj2∥ϕ∥2

Since the graph Laplacian matrix is symmetric, we can combine (i,j) and (j,i) together:

$$\begin{array}{cc} \; & \phi_{i}\phi_{j}^{3}-\phi_{i}^{2}\phi_{j}^{2}+\phi_{j}\phi_{i}^{3}-\phi_{i}^{2}\phi_{j}^{2}\\ \; & =\phi_{i}\phi_{j}^{3}+\phi_{j}\phi_{i}^{3}-2\phi_{i}^{2}\phi_{j}^{2}\\ \; & =\phi_{i}\phi_{j}\left(\phi_{i}^{2}+\phi_{j}^{2}-2\phi_{i}\phi_{j}\right)=\phi_{i}\phi_{j}{\left(\phi_{i}-\phi_{j}\right)}^{2} \end{array}$$

### 3.13. Detail Derivation of Training Loss in Equation (16)

We start with the derivation of the evidence lower bound:

logp(xt,yt|f,x0,β)≥Elogp(xt,yt,ϕ|f,x0,β)q(logϕ)=Eq(logϕ)logp(xt|ϕ,f,x0,β)q(yt|ϕ,xt,x0)−DKLq(logϕ)∥p(logϕ|Σ)−DKLq(logϕ)∥q(logϕ)≈1M∑k=1M−∥xt−f(t|x0,β,Lk)∥2+∥yt−(ϕk⊤xt)2ϕk⊤ϕk∥2︸ReconstructionLoss−12TrΣ−1ΨΨ⊤+νΣ−1ν+log1det(ΨΨ⊤)−N︸KLDivergence

where the log-determinant is canceled because we took the diagonal as constant for Ψ.

### 3.14. Detail Derivation of the Linear Source Solution, Equation (1)

First, Equation (3) is an in-homogeneous ODEs system. The standard solution format is

$$x\left(t\right)=e^{-\beta tL}x_{0}+e^{-\beta tL}\int_{0}^{t}e^{\beta \tau L}r\tau d\tau$$

With the eigendecomposition given by $L=U\Lambda U^{⊤}$, we can factor out the parameters r from the integral and solve the integral by matrix integration in parts:

$$\begin{array}{cc} \; & =Ue^{-\beta t\Lambda }U^{⊤}x_{0}+Ue^{-\beta t\Lambda }U^{⊤}r\int_{0}^{t}e^{\beta \tau \Lambda }\tau d\tau \end{array}$$

Note that

$$e^{-\beta tL}=Ue^{-\beta t\Lambda }U^{⊤}$$

Inside the integral, if Λ is invertible, we have

$$\begin{array}{cc} \; & \int_{0}^{t}e^{\beta \tau L\Lambda }\tau d\tau ={\left[\frac{1}{\beta \Lambda }e^{\beta \tau \Lambda }\right]}_{0}^{t}-\frac{1}{\beta \Lambda }\int_{0}^{t}e^{\beta \tau \Lambda }d\tau \\ \; & =\frac{1}{\beta \Lambda }e^{\beta \tau \Lambda }-\frac{1}{\beta^{2}\Lambda^{2}}\left(e^{\beta \tau \Lambda }-I\right) \end{array}$$

However, as per Theorem 1, we know the smallest eigenvalue is 0. We therefore approximate the diagonal matrix by the largest eigenvalue, which is

$$\left(\frac{1}{\beta \lambda_{1}}e_{1}^{\beta t\lambda }-\frac{1}{\beta^{2}\lambda_{1}^{2}}\left(e_{1}^{\beta t\lambda }-1\right)\right)$$

### 3.15. Detailed Derivation of the Exponential Source Solution, Equation (2)

Similarly, we have the solution format in if Λ is invertible:

$$\begin{array}{cc} \; & x\left(t\right)=e^{-\beta tL}x_{0}+e^{-\beta tL}\int_{0}^{t}e^{\beta \tau L}a\left(e^{\xi \tau }-1\right)\tau d\tau \\ \; & =Ue^{-\beta t\Lambda }U^{⊤}x_{0}+Ue^{-\beta t\Lambda }U^{⊤}a\int_{0}^{t}e^{\beta \tau \Lambda }\left(e^{\xi \tau }-1\right)d\tau \\ \; & =Ue^{-\beta t\Lambda }U^{⊤}x_{0}+Ue^{-\beta t\Lambda }U^{⊤}a(\frac{1}{\beta \Lambda +\xi }(e^{t(\beta \Lambda +\xi )}-\\ \; & I)-\frac{1}{\beta \Lambda }\left(e^{\beta t\Lambda }-I\right)) \end{array}$$

Because there exists a zero diagonal of Λ, we conduct the same 1-D approximation, yielding

$$\left(\frac{1}{\beta \lambda_{1}+\xi }\left(e^{t(\beta \lambda_{1}+\xi )}-1\right)-\frac{1}{\beta \lambda_{1}}\left(e^{\beta t\lambda_{1}}-1\right)\right)$$

## 4. Conclusions

A Bayesian variational inference framework was developed that infers latent brain connectome changes in the development of neurological disorders when no longitudinal brain network data are provided. Brain networks were successfully estimated at different stages of disease. Experiments with real-world AD data showed an ability to differentiate normative aging from AD pathology. Additionally, results revealed novel evidence of the “decoupling” hypothesis and dynamic network mathematical instability in AD as calculated via eigenvalues. This framework lays a dynamic computational foundation for future personalized predictive medicine with a high societal impact.

## Figures and Tables

**Table 1:** The mean and the standard deviation of MSE of meta-models with different ACP mechanisms when fit to MCI and AD biomarkers. Each experiment was repeated 30 times.

ACP + Connectome Windows	ADNI-AV45-PET	Synthetic-AV45	ADNI-1451-PET
No Source 1-Windows	0.00022±0.00026	1.35±0.92	1.26±1.92
No Source 2-Windows	0.00020±0.00019	1.35±0.92	1.25±1.92
Linear Source 1-Windows	0.019±0.034	0.46±0.93	1.84±0.44
Linear Source 2-Windows	0.015±0.014	0.45±0.60	1.72±0.43
Exponential Source 1-Windows	0.017±0.020	0.50±0.40	1.83±1.61
Exponential Source 2-Windows	0.016±0.013	0.60±0.80	1.89±1.47

Bold numbers are the best out of the experiments of the entire dataset.

**Table 2:** The Pearson correlation coefficients between actual and predicted outputs of the optimal meta-model regression results shown in Figure 7.

Pearson Coefficient	Early	Late
Hippocampus	0.36 ***	0.012
Ventricles	0.99 ***	0.96 ***
Entorhinal	0.31 ***	0.03

*** is the significance level of 1%.

**Table 3:** The precision of identifying the decoupling subcortical regions by selecting the first/largest 10 values from Synthetic-1451-PET. The experiments were repeated 30 times. GP is not available because its decaying rate is handcrafted and pre-determined.

Model	Precision
2-Windows	0.30±0.11
EBM	0.23±0.15
GP	NA

## Data Availability

Access to vae_connectome code: https://github.com/pathology-dynamics [accessed 28 December 2024]. Access to ADNI data sets must be requested from https://adni.loni.usc.edu/data-samples/ [accessed 28 December 2024].

## Abbreviations

The following abbreviations are used in this manuscript:

AD	Alzheimer’s Disease
MCI	Mild Cognitive Impairment
H	Healthy
APP	Amyloid Precursor Protein
Aβ, A-beta	Amyloid Beta Protein
τ, tau	Tau Misfolded Protein
NFTs	Neurofibrillary Tangles
IVP	Initial Value Problem
ACP	Accumulation, Clearance, Propagation
PET	Positive Emission Tomography
MRI	Magnetic Resonance Imaging
MSE	Mean Squared Error
ODE	Ordinary Differential Equations
EBM	Event-based Models
GP	Gaussian Processes
ADNI	Alzheimer’s Disease Neuroimaging Initiative

## References

[1] Anita Pothen S. (2022). The Economic and Societal Burden of Alzheimer Disease: Managed Care Considerations. Am. J. Manag. Care.

[2] Scheltens P., De Strooper B., Kivipelto M., Holstege H., Chételat G., Teunissen C.E., Cummings J., van der Flier W.M. (2021). Alzheimer’s disease. Lancet.

[3] Gulisano W., Maugeri D., Baltrons M.A., Fà M., Amato A., Palmeri A., D’Adamio L., Grassi C., Devanand D., Honig L.S. (2018). Role of Amyloid-*β* and Tau Proteins in Alzheimer’s Disease: Confuting the Amyloid Cascade. J. Alzheimer’s Dis..

[4] Kumar V., Kim S.H., Bishayee K. (2022). Dysfunctional Glucose Metabolism in Alzheimer’s Disease Onset and Potential Pharmacological Interventions. Int. J. Mol. Sci..

[5] O’Brien R.J., Wong P.C. (2011). Amyloid Precursor Protein Processing and Alzheimer’s Disease. Annu. Rev. Neurosci..

[6] Chen G.F., Xu T.H., Yan Y., Zhou Y.R., Jiang Y., Melcher K., Xu H.E. (2017). Amyloid beta: Structure, biology and structure-based therapeutic development. Acta Pharmacol. Sin..

[7] Kinney J.W., Bemiller S.M., Murtishaw A.S., Leisgang A.M., Salazar A.M., Lamb B.T. (2018). Inflammation as a central mechanism in Alzheimer’s disease. Alzheimer’s Dement. Transl. Res. Clin. Interv..

[8] Venegas C., Kumar S., Franklin B.S., Dierkes T., Brinkschulte R., Tejera D., Vieira-Saecker A., Schwartz S., Santarelli F., Kummer M.P. (2017). Microglia-derived ASC specks cross-seed amyloid-*β* in Alzheimer’s disease. Nature.

[9] Zotova E., Nicoll J.A., Kalaria R., Holmes C., Boche D. (2010). Inflammation in Alzheimer’s disease: Relevance to pathogenesis and therapy. Alzheimer’s Res. Ther..

[10] Alonso A.d.C., Li B., Grundke-Iqbal I., Iqbal K. (2006). Polymerization of hyperphosphorylated tau into filaments eliminates its inhibitory activity. Proc. Natl. Acad. Sci. USA.

[11] Barbier P., Zejneli O., Martinho M., Lasorsa A., Belle V., Smet-Nocca C., Tsvetkov P.O., Devred F., Landrieu I. (2019). Role of Tau as a Microtubule-Associated Protein: Structural and Functional Aspects. Front. Aging Neurosci..

[12] Gong C.X., Iqbal K. (2008). Hyperphosphorylation of Microtubule-Associated Protein Tau: A Promising Therapeutic Target for Alzheimer Disease. Curr. Med. Chem..

[13] Zhang H., Wei W., Zhao M., Ma L., Jiang X., Pei H., Cao Y., Li H. (2021). Interaction between **A***β* and Tau in the Pathogenesis of Alzheimer’s Disease. Int. J. Biol. Sci..

[14] Malkov A., Popova I., Ivanov A., Jang S.S., Yoon S.Y., Osypov A., Huang Y., Zilberter Y., Zilberter M. (2021). **A***β* initiates brain hypometabolism, network dysfunction and behavioral abnormalities via NOX2-induced oxidative stress in mice. Commun. Biol..

[15] Gallego-Rudolf J., Wiesman A.I., Pichet Binette A., Villeneuve S., Baillet S. (2024). Synergistic association of **A***β* and tau pathology with cortical neurophysiology and cognitive decline in asymptomatic older adults. Nat. Neurosci..

[16] Zhang J., Zhang Y., Wang J., Xia Y., Zhang J., Chen L. (2024). Recent advances in Alzheimer’s disease: Mechanisms, clinical trials and new drug development strategies. Signal Transduct. Target. Ther..

[17] Kamatham P.T., Shukla R., Khatri D.K., Vora L.K. (2024). Pathogenesis, diagnostics, and therapeutics for Alzheimer’s disease: Breaking the memory barrier. Ageing Res. Rev..

[18] Jucker M., Walker L.C. (2011). Pathogenic protein seeding in Alzheimer disease and other neurodegenerative disorders. Ann. Neurol..

[19] Sheline Y.I., Raichle M.E., Snyder A.Z., Morris J.C., Head D., Wang S., Mintun M.A. (2010). Amyloid plaques disrupt resting state default mode network connectivity in cognitively normal elderly. Biol. Psychiatry.

[20] Frere S., Slutsky I. (2018). Alzheimer’s Disease: From Firing Instability to Homeostasis Network Collapse. Neuron.

[21] Iadecola C., Smith E.E., Anrather J., Gu C., Mishra A., Misra S., Perez-Pinzon M.A., Shih A.Y., Sorond F.A., van Veluw S.J. (2023). The Neurovasculome: Key Roles in Brain Health and Cognitive Impairment: A Scientific Statement From the American Heart Association/American Stroke Association. Stroke.

[22] Ali D.G., Bahrani A.A., Barber J.M., El Khouli R.H., Gold B.T., Harp J.P., Jiang Y., Wilcock D.M., Jicha G.A. (2022). Amyloid-PET Levels in the Precuneus and Posterior Cingulate Cortices Are Associated with Executive Function Scores in Preclinical Alzheimer’s Disease Prior to Overt Global Amyloid Positivity. J. Alzheimer’s Dis..

[23] Tastan B., Heneka M.T. (2024). The impact of neuroinflammation on neuronal integrity. Immunol. Rev..

[24] Xu J., Song W., Xu Z., Danziger M.M., Karavani E., Zang C., Chen X., Li Y., Paz I.M.R., Gohel D. (2024). Single-microglia transcriptomic transition network-based prediction and real-world patient data validation identifies ketorolac as a repurposable drug for Alzheimer’s disease. Alzheimer’s Dement..

[25] Buckner R.L., Sepulcre J., Talukdar T., Krienen F.M., Liu H., Hedden T., Andrews-Hanna J.R., Sperling R.A., Johnson K.A. (2009). Cortical Hubs Revealed by Intrinsic Functional Connectivity: Mapping, Assessment of Stability, and Relation to Alzheimer’s Disease. J. Neurosci..

[26] Kang S., Lee Y.H., Lee J.E. (2017). Metabolism-Centric Overview of the Pathogenesis of Alzheimer’s Disease. Yonsei Med. J..

[27] Vemuri P., Jack C.R. (2010). Role of structural MRI in Alzheimer’s disease. Alzheimer’s Res. Ther..

[28] Li Y., Liu T., Zeng Q., Cui M. (2024). Current status of PET tracers for the early diagnosis of Alzheimer’s disease. TrAC Trends Anal. Chem..

[29] Bao W., Xie F., Zuo C., Guan Y., Huang Y.H. (2021). PET Neuroimaging of Alzheimer’s Disease: Radiotracers and Their Utility in Clinical Research. Front. Aging Neurosci..

[30] Garbarino S., Lorenzi M., Initiative A.D.N. (2019). Modeling and inference of spatio-temporal protein dynamics across brain networks. Proceedings of the Information Processing in Medical Imaging: 26th International Conference, IPMI 2019.

[31] Schäfer A., Mormino E.C., Kuhl E. (2020). Network diffusion modeling explains longitudinal tau pet data. Front. Neurosci..

[32] Yang F., Chowdhury S.R., Jacobs H.I., Sepulcre J., Wedeen V.J., Johnson K.A., Dutta J. (2021). Longitudinal predictive modeling of tau progression along the structural connectome. Neuroimage.

[33] Raj A., Kuceyeski A., Weiner M. (2012). A network diffusion model of disease progression in dementia. Neuron.

[34] Fornari S., Schäfer A., Jucker M., Goriely A., Kuhl E. (2019). Prion-like spreading of Alzheimer’s disease within the brain’s connectome. J. R. Soc. Interface.

[35] Garbarino S., Lorenzi M., For the Alzheimer’s Disease Neuroimaging Initiative (2021). Investigating hypotheses of neurodegeneration by learning dynamical systems of protein propagation in the brain. NeuroImage.

[36] Iturria-Medina Y., Sotero R.C., Toussaint P.J., Evans A.C., Initiative A.D.N. (2014). Epidemic spreading model to characterize misfolded proteins propagation in aging and associated neurodegenerative disorders. PLoS Comput. Biol..

[37] Vogel J.W., Iturria-Medina Y., Strandberg O.T., Smith R., Levitis E., Evans A.C., Hansson O. (2020). Spread of pathological tau proteins through communicating neurons in human Alzheimer’s disease. Nat. Commun..

[38] Oxtoby N.P., Garbarino S., Firth N.C., Warren J.D., Schott J.M., Alexander D.C., Initiative A.D.N. (2017). Data-driven sequence of changes to anatomical brain connectivity in sporadic Alzheimer’s disease. Front. Neurol..

[39] Thompson E., Schroder A., He T., Shand C., Soskic S., Oxtoby N.P., Barkhof F., Alexander D.C., Initiative A.D.N. (2024). Combining multimodal connectivity information improves modelling of pathology spread in Alzheimer’s disease. Imaging Neurosci..

[40] Tandon R., Kirkpatrick A., Mitchell C.S. (2023). sEBM: Scaling event based models to predict disease progression via implicit biomarker selection and clustering. Proceedings of the International Conference on Information Processing in Medical Imaging.

[41] Tandon R., Lah J.J., Mitchell C.S., Pollard T., Choi E., Singhal P., Hughes M., Sizikova E., Mortazavi B., Chen I., Wang F., Sarker T., McDermott M. (2024). s-SuStaIn: Scaling subtype and stage inference via simultaneous clustering of subjects and biomarkers. Proceedings of the Fifth Conference on Health, Inference, and Learning.

[42] Lorenzi M., Filippone M. Constraining the dynamics of deep probabilistic models. Proceedings of the International Conference on Machine Learning.

[43] Bhaskar D., Magruder D.S., Morales M., De Brouwer E., Venkat A., Wenkel F., Wolf G., Krishnaswamy S. Inferring dynamic regulatory interaction graphs from time series data with perturbations. Proceedings of the Learning on Graphs Conference.

[44] Salvadó G., Horie K., Barthélemy N.R., Vogel J.W., Pichet Binette A., Chen C.D., Aschenbrenner A.J., Gordon B.A., Benzinger T.L., Holtzman D.M. (2024). Disease staging of Alzheimer’s disease using a CSF-based biomarker model. Nat. Aging.

[45] van der Velpen I.F., Vlasov V., Evans T.E., Ikram M.K., Gutman B.A., Roshchupkin G.V., Adams H.H., Vernooij M.W., Ikram M.A. (2023). Subcortical brain structures and the risk of dementia in the Rotterdam Study. Alzheimer’s Dement..

[46] de Jong L.W., van der Hiele K., Veer I.M., Houwing J.J., Westendorp R.G.J., Bollen E.L.E.M., de Bruin P.W., Middelkoop H.A.M., van Buchem M.A., van der Grond J. (2008). Strongly reduced volumes of putamen and thalamus in Alzheimer’s disease: An MRI study. Brain.

[47] de Haan W., Mott K., van Straaten E.C.W., Scheltens P., Stam C.J. (2012). Activity Dependent Degeneration Explains Hub Vulnerability in Alzheimer’s Disease. PLoS Comput. Biol..

[48] Mitchell C.S., Lee R.H. (2008). Pathology dynamics predict spinal cord injury therapeutic success. J. Neurotrauma.

[49] Irvin C.W., Kim R.B., Mitchell C.S. (2015). Seeking homeostasis: Temporal trends in respiration, oxidation, and calcium in SOD1 G93A Amyotrophic Lateral Sclerosis mice. Front. Cell. Neurosci..

[50] Lee A.J.B., Bi S., Ridgeway E., Al-Hussaini I., Deshpande S., Krueger A., Khatri A., Tsui D., Deng J., Mitchell C.S. (2025). Restoring Homeostasis: Treating Amyotrophic Lateral Sclerosis by Resolving Dynamic Regulatory Instability. Int. J. Mol. Sci..

[51] Tandon R., Levey A.I., Lah J.J., Seyfried N.T., Mitchell C.S. (2023). Machine learning selection of most predictive brain proteins suggests role of sugar metabolism in Alzheimer’s disease. J. Alzheimers. Dis..

[52] Serra L., Cercignani M., Mastropasqua C., Torso M., Spanò B., Makovac E., Viola V., Giulietti G., Marra C., Caltagirone C. (2016). Longitudinal changes in functional brain connectivity predicts conversion to Alzheimer’s disease. J. Alzheimer’s Dis..

[53] Seeley W.W., Crawford R.K., Zhou J., Miller B.L., Greicius M.D. (2009). Neurodegenerative diseases target large-scale human brain networks. Neuron.

[54] Delbeuck X., Van der Linden M., Collette F. (2003). Alzheimer’disease as a disconnection syndrome?. Neuropsychol. Rev..

[55] Crossley N.A., Mechelli A., Scott J., Carletti F., Fox P.T., McGuire P., Bullmore E.T. (2014). The hubs of the human connectome are generally implicated in the anatomy of brain disorders. Brain.

[56] Warren J.D., Rohrer J.D., Schott J.M., Fox N.C., Hardy J., Rossor M.N. (2013). Molecular nexopathies: A new paradigm of neurodegenerative disease. Trends Neurosci..

[57] Maleki Balajoo S., Rahmani F., Khosrowabadi R., Meng C., Eickhoff S.B., Grimmer T., Zarei M., Drzezga A., Sorg C., Tahmasian M. (2022). Decoupling of regional neural activity and inter-regional functional connectivity in Alzheimer’s disease: A simultaneous PET/MR study. Eur. J. Nucl. Med. Mol. Imaging.

[58] Alster P., Krzyżanowska E., Koziorowski D., Szlufik S., Różański D., Noskowska J., Mianowicz J., Michno A., Królicki L., Friedman A. (2018). Difficulties in the diagnosis of four repeats (4R) tauopathic parkinsonian syndromes. Neurol. Neurochir. Pol..

[59] Spielman D. (2012). Spectral graph theory. Comb. Sci. Comput..

[60] Agarwal R.P., Agarwal R.P., Lakshmikantham V. (1993). Uniqueness and Nonuniqueness Criteria for Ordinary Differential Equations.

